# Improvement of multilineage hematopoiesis in hematopoietic stem cell-transferred c-kit mutant NOG-EXL humanized mice

**DOI:** 10.1186/s13287-024-03799-w

**Published:** 2024-06-21

**Authors:** Ryoji Ito, Yusuke Ohno, Yunmei Mu, Yuyo Ka, Shuko Ito, Maiko Emi-Sugie, Misa Mochizuki, Kenji Kawai, Motohito Goto, Tomoyuki Ogura, Riichi Takahashi, Akira Niwa, Tatsutoshi Nakahata, Mamoru Ito

**Affiliations:** 1 Central Institute for Experimental Medicine and Life Science, Kawasaki, Kanagawa 210-0821 Japan; 2 Department of Immunology, Mayo Clinic College of Medicine and Science, Rochester, MN 55905 USA; 3 Department of Clinical Application, Center for iPS Cell Research and Application, Kyoto University, Kyoto, 606-8507 Japan

**Keywords:** Hematopoietic stem cells, Hematopoiesis, Humanized mice, NOG, CD34^+^ cells

## Abstract

Human hematopoietic stem cell (HSC)-transferred humanized mice are valuable models for exploring human hematology and immunology. However, sufficient recapitulation of human hematopoiesis in mice requires large quantities of enriched human CD34^+^ HSCs and total-body irradiation for adequate engraftment. Recently, we generated a NOG mouse strain with a point mutation in the c-kit tyrosine kinase domain (W41 mutant; NOGW mice). In this study, we examined the ability of NOGW mice to reconstitute human hematopoietic cells. Irradiated NOGW mice exhibited high engraftment levels of human CD45^+^ cells in the peripheral blood, even when only 5,000–10,000 CD34^+^ HSCs were transferred. Efficient engraftment of human CD45^+^ cells was also observed in non-irradiated NOGW mice transferred with 20,000–40,000 HSCs. The bone marrow (BM) of NOGW mice exhibited significantly more engrafted human HSCs or progenitor cells (CD34^+^CD38^−^ or CD34^+^CD38^+^ cells) than the BM of NOG mice. Furthermore, we generated a human cytokine (interleukin-3 and granulocyte-macrophage colony-stimulating factor) transgenic NOG-W41 (NOGW-EXL) mouse to achieve multilineage reconstitution with sufficient engraftment of human hematopoietic cells. Non-irradiated NOGW-EXL mice showed significantly higher engraftment levels of human CD45^+^ and myeloid lineage cells, particularly granulocytes and platelets/megakaryocytes, than non-irradiated NOGW or irradiated NOG-EXL mice after human CD34^+^ cell transplantation. Serial BM transplantation experiments revealed that NOGW mice exhibited the highest potential for long-term HSC compared with other strains. Consequently, c-kit mutant NOGW-EXL humanized mice represent an advanced model for HSC-transferred humanized mice and hold promise for widespread applications owing to their high versatility.

**Supplementary Information:**

The online version contains supplementary material available at 10.1186/s13287-024-03799-w.

## Significance statement

Humanized mice engrafted with human hematopoietic stem cells (HSCs) can reconstitute the human hematologic environment and are valuable in hematology and immunology research. Standard methods require large numbers of enriched CD34^+^ HSCs and whole-body irradiation to achieve sufficient engraftment in humanized mice. Here, we examined human hematopoietic cell reconstitution in a NOG mouse strain with a point mutation in the c-kit tyrosine kinase domain (NOGW and NOGW-EXL mice). Our findings revealed that NOGW and NOGW-EXL mice present an irradiation-free approach for the efficient engraftment of multilineage human hematopoietic cells and require a reduced number of CD34^+^ HSCs for transplantation.

**Supplementary Information:**

The online version contains supplementary material available at 10.1186/s13287-024-03799-w.

## Introduction

Reconstitution of the human hematologic environment in severely immunodeficient mice plays a pivotal role in advancing the field of humanized mouse research, which is valuable for investigations pertaining to human hematology and immunology. Significant challenges in this field include achieving a high engraftment capacity and multi lineage differentiation of human hematopoietic cells within humanized mice in order to create models that accurately mimic human hematopoietic processes. NOD-*scid* IL-2rg^*null*^ (NOG and NSG) mice possess a mutated *Prkdc* gene (severe combined immunodeficiency; scid) and lack the interleukin (IL)-2 receptor subunit gamma (IL-2rg), making them useful recipients for transferring CD34^+^ human hematopoietic stem cells (HSCs) [1, 2]. The high engraftment rates observed in NOG and NSG mice are attributed to the deficiency of T cells, B cells, and natural killer (NK) cells as well as to the enhanced engraftment of human cells resulting from the specific *Sirpa* polymorphism in NOD background mice [3]. We previously established NOG-based second-generation humanized mice that systemically express myeloid cell–accelerated cytokines and successfully induce the differentiation of multiple human myeloid lineage cells, such as monocytes/macrophages, basophils, mast cells [4], eosinophils [5], and neutrophils [6]. To achieve sufficient human hematopoietic cell engraftment in humanized mice, standard methods require a large number of enriched CD34^+^ HSCs along with total body irradiation before transplantation. Human hematopoietic cells can be engrafted into immunodeficient NSG or NOD/B6-*scid* IL-2rg^*null*^ mice with a point mutation in the c-kit tyrosine kinase domain without X-ray irradiation [7, 8]. Compared to conventional humanized mice, the bone marrow (BM) of c-kit-mutated immunodeficient mice showed substantial maintenance of CD34^+^ human HSCs. Furthermore, a high engraftment rate of human hematopoietic cells was observed in their periphery. Recently, we reported that the W41 mutant NOG (NOGW) mice exhibit high engraftment of human leukocytes without X-ray irradiation. We successfully detected human CD45^+^ cells in the peripheral blood (PB), BM, and spleen of NOGW mice, transferred with in vitro-expanded CD34^+^ cells derived from a single cell, using a chemically defined expansion medium for human HSCs [9]. Therefore, the W41 mutation in immunodeficient mice can improve the engraftment capacity of human hematopoietic cells and may enable successful engraftment by transferring a small number of human CD34^+^ cells. However, in the current W41 strain, human myeloid lineage cells still do not fully differentiate because the human myeloid cell-accelerated cytokines, such as IL-3 and GM-CSF have not been introduced.

This study aimed to examine the engraftment capacity of human hematopoietic cells in NOGW mice using the limiting dilution assay and compare the engraftment and differentiation potentials of human hematopoietic and progenitor cells in NOG, NOG-hIL-3/granulocyte-macrophage colony-stimulating factor (GM-CSF) Tg (EXL), NOGW, and NOGW-EXL mice.

## Materials and methods

### Mice

NOG [1] (NOD.Cg-*prkdc*^*scid*^*il2rg*^*tm1Sug*^*/*Jic) and NOG-EXL [4] (NOG-hIL-3/GM-CSF Tg; NOD.Cg-*Prkdc*^*scid*^*Il2rg*^*tm1Sug*^*Tg (SRa-IL3, CSF2)*/Jic) mice were previously established at the Central Institute for Experimental Medicine and Life Science (CIEM). NOGW (NOD.Cg-*Prkdc*^*scid*^*Il2rg*^*tm1Sug*^*Kit*^*em1(V831M)*^Jic) mice were established by genome editing using the transcription activator-like effector nuclease technique [9]. NOG-EXL mice were backcrossed with NOGW mice to produce NOGW-EXL mice. The mice had access to sterilized food and water *ad libitum* and were used for human cell transplantation studies at 6–12 weeks of age.

### Generation of humanized mice

X-ray irradiation was performed using the MBR-1520R-4 model system (Hitachi Power Solutions Co., Ltd., Ibaraki, Japan) to generate humanized mice. Commercially available human cord blood CD34^+^ cells, purchased from StemExpress LLC (Folsom, CA, USA), were used; the cells were prepared according to the manufacturer’s instructions. Briefly, cryopreserved CD34^+^ cell vials were thawed in a water bath at 37 °C and immediately transferred to RPMI1640 medium (Thermo Fisher Scientific, Waltham, MA, USA), containing 10% fetal calf serum and DNase I (Roche Diagnostics, Basel, Switzerland). The viability of CD34^+^ HSCs was measured using 2.5% trypan blue staining, and cells with > 90% viability were used for transplantation. Subsequently, these human HSCs were intravenously injected into 1.5-Gy-irradiated NOG and NOG-EXL mice, non-irradiated or 1-Gy-irradiated NOGW mice, and non-irradiated NOGW-EXL mice.

### Flow cytometry

Human or mouse immune cells in the PB, BM, and spleen were analyzed using flow cytometry and stained with anti-human or anti-mouse antibodies. The total cell number in the tissues was determined using a Microsemi LC662 hematology analyzer (HORIBA Ltd., Kyoto, Japan). Cells were prepared using BD Pharm-Lyse (BD Biosciences, San Jose, CA, USA) to remove red blood cells and incubated in the dark for 20 min at 4 °C with a mixture of fluorescently labeled monoclonal antibodies. After washing with phosphate-buffered saline, the cells were suspended in propidium iodide/RNase staining buffer (BD Biosciences), subjected to multicolor flow cytometry with the LSR Fortessa X-20 instrument (BD Biosciences), and analyzed using FlowJo version 10.6.2 software (BD Biosciences). The engraftment ratio of human cells was expressed as the percentage of human CD45^+^ cells relative to the total number of leukocytes (mouse and human), excluding erythrocytes and/or debris. Details of the antibodies against the cell surface molecules are provided in Table S1.

### ELISA

The PB and BM samples were collected from 8- to 12-weeks-old male/female NOG or NOGW mice, and the plasma and BM fluid were harvested after centrifugation. Murine stem cell factor (SCF) levels were measured by using mouse stem cell factor Enzyme-Linked Immunosorbent Assay Kits (R&D Systems, Minneapolis, MN), as per the manufacturer’s instructions.

### Serial BM transplantation

Briefly, 1 × 10^7^ BM cells were isolated from the femurs of humanized mice at 16 weeks, reconstituted with 2.5 × 10^4^ human CD34^+^ cells, and serially transplanted into 1.5-Gy-irradiated NOG mice via the tail vein. The frequency of engrafted human hematopoietic cells in PB and BM was analyzed using flow cytometry.

### Immunohistochemistry

Bone, liver, and lung tissues were harvested, fixed overnight in 10% buffered formalin (FujiFilm, Wako, Osaka, Japan), and embedded in paraffin. Tissue Sect. (3-µm thick) were placed on aminosilane-coated glass slides (Muto Pure Chemicals, Tokyo, Japan) and immunostained using a Bond-Max automated stainer and a BOND Polymer Refine Detection system (Leica Biosystems K.K., Tokyo, Japan). After deparaffinization, the sections were incubated with an anti-human CD61 antibody (clone Y2/51) (Agilent Technologies Inc., Santa Clara, CA, USA) for 30 min, and then incubated with the polymer for 30 min, followed by DAB chromogen for 10 min. To visualize the nuclei, the immunostained sections were counterstained with hematoxylin (Leica Biosystems K. K.).

### Statistical analyses

Data are presented in terms of the means ± standard deviations. The significance of the differences was evaluated using a two-tailed Student’s *t*-tests or one-way analysis of variance. Statistical analyses were performed using Excel (Microsoft Corp., Redmond, WA, USA) or GraphPad Prism 7 (GraphPad, San Diego, CA, USA). A *p*-value of < 0.05 was considered statistically significant. Statistical details are shown in the respective figure legends.

## Results and discussion

### Human hematopoiesis in NOGW mice

**Fig. 1:**
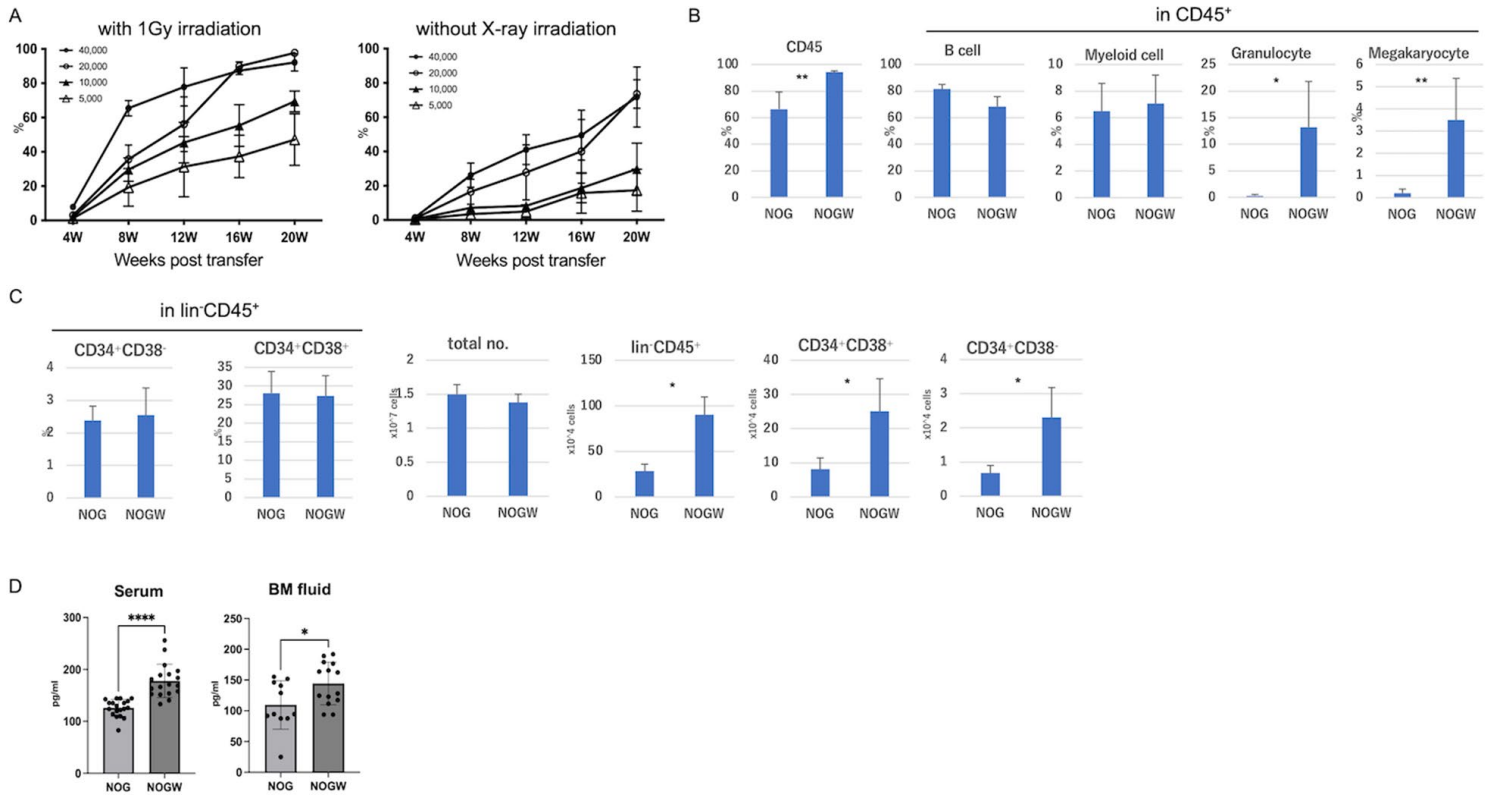
Human hematopoiesis in NOGW mice. **(A)** HSC-limiting dilution assay in NOGW mice. Either 40,000, 20,000, 10,000, or 5,000 CD34^+^ HSCs were transferred into 1-Gy-irradiated or non-irradiated NOGW mice (*n* = 4–5). The chimeric ratio of human CD45^+^ cells in the PB was analyzed by flow cytometry at indicated time points. **(B)** Frequencies of human CD45^+^ cells, CD19^+^ B cells, CD33^+^ myeloid cells, CD66b^+^ granulocytes, and CD41^+^ megakaryocytes in the BM of humanized NOG and NOGW mice 16 weeks after HSC transplantation (*n* = 5). **(C)** Frequencies of human CD34^+^CD38^−^ cells and CD34^+^CD38^+^ cells in lineage^−^CD45^+^ cells, and number of total leukocytes, lineage^−^CD45^+^ cells, CD34^+^CD38^−^ cells, and CD34^+^CD38^+^ cells in BM of humanized NOG or NOGW mice. **(D)** Mouse SCF protein levels in serum (NOG; *n* = 19, NOGW; *n* = 18) or BM fluid (NOG; *n* = 13, NOGW; *n* = 22) of non-humanized NOG or NOGW mice. ^*^p < 0.05; ^**^p < 0.01; ^****^p < 0.001

Typically, a high number of human CD34^+^ cells is required to achieve sufficient engraftment of human hematopoietic cells in various organs of immunodeficient mice via total-body irradiation during humanized mouse generation [10–12]. Lang et al. reported that at least 50,000 umbilical cord blood-derived human CD34^+^ cells were required to reconstitute an adequate population of human hematopoietic cells in irradiated BALB/c-Rag2^*null*^ Il2rg^*null*^ mice [13]. However, the optimal number of CD34^+^ cells varies depending on the specific immunodeficient mouse strain, indicating a strain-dependent variability in the engraftment capacity of human hematopoietic cells. In this study, we aimed to identify the optimal number of CD34^+^ HSCs for reconstituting human hematopoiesis in NOGW mice, with or without total-body irradiation. Initially, we assessed the sensitivity of NOGW mice to irradiation without transplantation. The survival ratios of NOGW mice up to a 20-day period was 69% for females and 57% for males after exposure to 1.5 Gy irradiation (Figure S1). In contrast, most NOG mice survived at the same irradiation dose. However, all NOGW mice survived exposure to 1 Gy irradiation. Therefore, 1 Gy was determined to be the optimal irradiation dose for the NOGW strain. Irradiated NOGW mice exhibited sufficient engraftment of human CD45^+^ cells into PB after the transfer of at least 10,000 CD34^+^ cells. Efficient hematopoiesis was also observed in non-irradiated NOGW mice following the transfer of at least 20,000 CD34^+^ cells (Fig. 1A). Subsequently, we compared human hematopoiesis in the BM of irradiated NOG and non-irradiated NOGW mice. The frequency of human CD45^+^ cells was significantly higher in the BM of NOGW mice than in that of NOG mice (Fig. 1B). In addition to the high frequency of hCD45^+^ cell engraftment, the BM of NOGW mice showed a marked increase in hCD66b^+^ granulocytes and hCD41^+^ megakaryocytes compared to the BM of NOG mice (Fig. 1B). Although the frequencies of CD34^+^CD38^+^ and CD34^+^CD38^−^ cells in the lineage (lin)-negative cell population remained unaltered in the BM of NOG and NOGW mice, the cell numbers of these populations were significantly higher in NOGW mice than in NOG mice (Fig. 1C). Stem cell factor (SCF), also known as the c-kit ligand, plays an essential role in promoting self-renewal and development and maintenance of HSC in the BM [14]. Owing to the high homology between humans and rodents, murine SCF binds to human c-kit and may promote the self-renewal of human HSC [15]. We compared the protein levels of murine SCF in the serum and BM fluid of NOG and NOGW mice without humanization. NOGW mice exhibited significantly higher levels of murine SCF in both serum and BM fluids than NOG mice (Fig. 1D). This observation suggests that murine SCF remains present in the blood and BM fluid, given its inability to bind to mutant c-kit. Importantly, these findings indicate that NOGW mice may have a greater potential for human cell engraftment, supporting the sustained presence of HSC or progenitor cells. Therefore, similar to previously established W41 strains [7, 8], the NOGW mouse model offers an irradiation-free approach to achieve efficient engraftment of human hematopoietic cells and reduces the number of CD34^+^ HSCs required for transplantation.

### Generation of NOGW-EXL mice

**Fig. 2:**
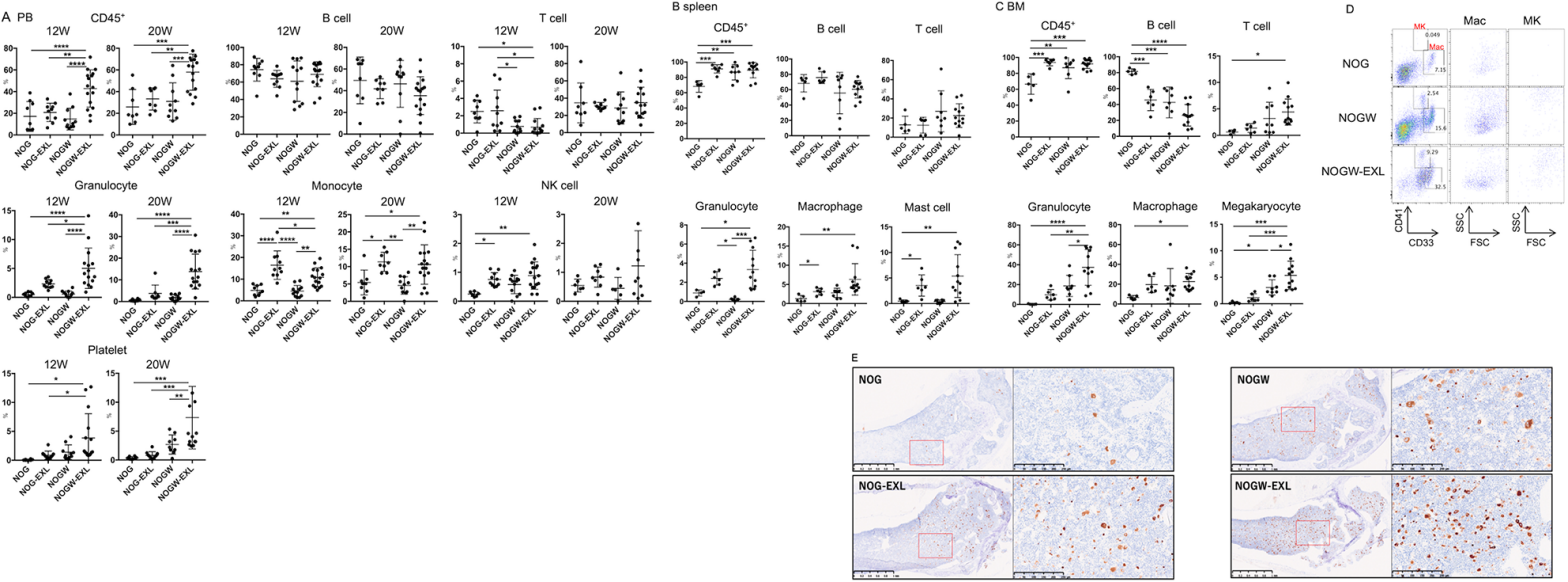
Generation of NOGW-EXL mice. **(A)** Frequencies of human CD45^+^ cells, CD19^+^ B cells, CD3^+^ T cells, CD66b^+^ granulocytes, CD33^+^14^+^ monocytes, CD56^+^ NK cells, and CD41^+^ platelets in the PB of humanized NOG, NOG-EXL, NOGW, and NOGW-EXL mice at 12 or 20 weeks after HSC transplantation (*n* = 6–16). **(B and C)** Frequencies of human CD45^+^ cells and other human cell lineages in the spleen **(B)** and BM **(C)** of humanized NOG, NOG-EXL, NOGW, and NOGW-EXL mice at 20 weeks after HSC transplantation (*n* = 6–16). **(D)** Representative flow cytometry analysis of human macrophages (Mac) and megakaryocytes (MK) in the BM of humanized NOG, NOGW, and NOGW-EXL mice 20 weeks after HSC transplantation. **(E)** Immunohistochemistry images of human CD61^+^ megakaryocytes in the bone tissue of humanized NOG, NOG-EXL, NOGW, and NOGW-EXL mice at 20 weeks after HSC transplantation. Each bone image is shown at low magnification (left) or high magnification (right) for each strain. Images are representative of three independent experiments. ^*^p < 0.05; ^**^p < 0.01; ^***^p < 0.005; ^****^p < 0.001

To achieve the differentiation of multiple cell lineages with advanced engraftment abilities, we backcrossed NOGW mice with NOG-EXL mice to generate NOGW-EXL mice. Then, we compared the reconstitution capacity of human hematopoietic cells in NOG, NOG-EXL, NOGW, and NOGW-EXL mice. At 12 and 20 weeks after CD34^+^ cell transplantation, non-irradiated NOGW-EXL mice exhibited higher rates of human CD45^+^ cell engraftment than irradiated NOG, NOG-EXL, and non-irradiated NOGW mice (Fig. 2A). NOGW-EXL mice showed higher reconstitution of human CD66b^+^ granulocytes, which are human eosinophils in most CD66^+^ cells [4], and CD41^+^ platelets than the other strains (Fig. 2A). Compared to NOG and NOGW mice, NOGW-EXL and NOG-EXL mice showed an increase in human CD33^+^14^+^ monocytes (Fig. 2A). We analyzed the sex-based differences in human cell engraftment in each strain of humanized mice. In most cases, no differences in human cell engraftment were observed between male and female mice. However, female NOGW-EXL and NOG-EXL mice exhibited significantly higher levels of CD45^+^ cells compared to their male counterparts at 12 and 20 weeks, respectively. This result is consisted with a previous report that the female NSG humanized mice show higher engraftment capacity of human CD45^+^ cells than male humanized mice [16]. In the spleen, the chimeric ratio of human CD45^+^ cells was higher in NOG-EXL, NOGW, and NOGW-EXL mice than that in conventional NOG mice, and total human myelopoiesis was significantly enhanced in NOG-EXL and NOGW-EXL mice (Fig. 2B). Tissue-resident human CD68^+^ macrophages exhibited significant engraftment in the liver and lungs of NOGW-EXL mice (Figures S3A and S3B). Next, we examined the development of human megakaryocytes in the NOGW-based strains. Previously, NSG-W41 and NBSGW mice displayed enhanced reconstitution of human megakaryocytes in the BM, with a detectable level of human platelets in PB [17, 18]. Similar to the previous W41 strain, NOGW mice displayed increased differentiation of human megakaryocytes compared to NOG mice. Among these strains, human megakaryocyte differentiation was significantly elevated in NOGW-EXL mice (Fig. 2C, D). Based on immunohistochemical analysis, the highest engraftment of human CD61^+^ megakaryocytes was observed in the BM of NOGW-EXL mice, which was further supported by flow cytometry studies (Fig. 2E). IL-3 plays a crucial role in megakaryopoiesis and platelet production in humans [19]. Considering the systemic production of human IL-3, NOGW-EXL mice plausibly exhibited accelerated human megakaryopoiesis compared with NOGW mice, leading to increased platelet production. The minimum number of HSCs required for humanization of non-irradiated NOGW-EXL mice was evaluated. Based on a limiting dilution assay of human HSCs, the transfer of 5,000 CD34^+^ HSCs achieved over 20% human CD45^+^ cell chimerism from 8 weeks onward (Figure S4). Additionally, the transfer of 10,000 HSCs yielded approximately 40% human CD45^+^ cell chimerism at 8 weeks in NOGW-EXL mice. The transfer of both 5,000 and 10,000 HSCs significantly reconstituted human granulocytes and platelets in NOGW-EXL mice but not NOGW mice (Figure S4). Thus, among the HSC-transferred humanized mouse models, the humanized NOGW-EXL mouse model exhibited greater hematopoiesis and engraftment capacity than NOG-EXL and NOGW mice.

### HSC maintenance in humanized mice

**Fig. 3:**
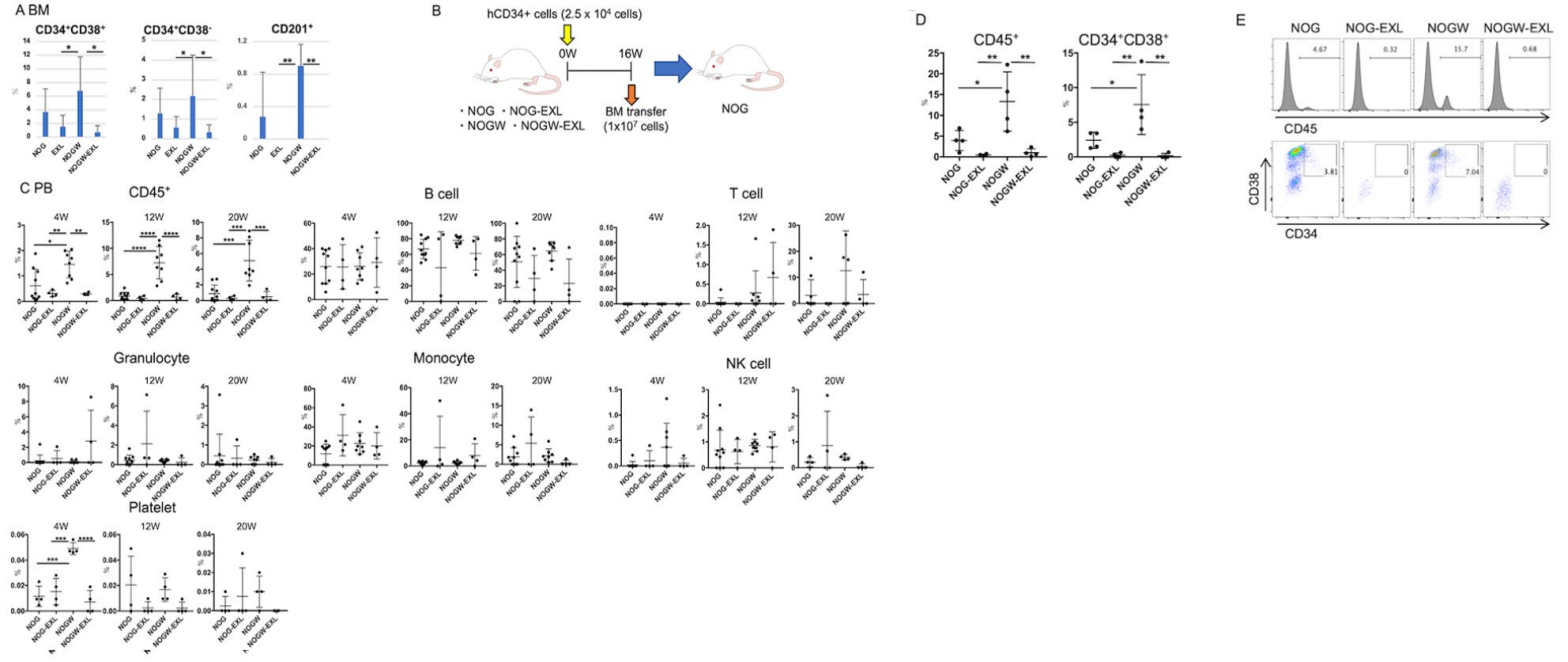
Maintenance of human HSCs in humanized mice. **(A)** Frequencies of human CD34^+^CD38^+^ cells and CD34^+^CD38^−^ cells in lineage^−^CD45^+^ cells and the frequency of CD201^+^ cells in CD34^+^CD38^−^ cells in the BM of humanized NOG, NOG-EXL, NOGW, and NOGW-EXL mice at 16 weeks after HSC transplantation. **(B)** Schema of the procedure for serial BM transplantation from humanized NOG, NOG-EXL, NOGW, and NOGW-EXL mice. **(C)** Frequencies of human CD45^+^ cells and other human lineage cells in the PB of secondary-recipient NOG mice after transfer of BM cells from humanized NOG, NOG-EXL, NOGW, and NOGW-EXL mice 4, 12, and 20 weeks after HSC transplantation (*n* = 4–8). **(D)** Frequencies of human CD45^+^ cells and CD34^+^CD38^+^ cells in the BM of secondary-recipient NOG mice 24 weeks after transfer of BM cells from humanized NOG, NOG-EXL, NOGW, and NOGW-EXL mice (*n* = 4). **(E)** Representative flow cytometric data of human CD45^+^ cells and CD34^+^CD38^+^ progenitor cells in D. ^*^p < 0.05; ^**^p < 0.01; ^***^p < 0.005; ^****^p < 0.001

We investigated the maintenance of human HSCs in various humanized mouse strains. In the flow cytometry analysis, the frequencies of human CD34^+^CD38^−^ multipotent hematopoietic progenitors and CD34^+^CD38^+^ hematopoietic progenitors in the BM of NOG-EXL and NOGW-EXL mice were significantly lower than those in the BM of NOGW mice (Fig. 3A). Additionally, CD201^+^, a critical marker of multipotent HSCs [20], was absent in CD34^+^CD38^−^ cells in NOG-EXL and NOGW-EXL mice (Fig. 3A). This finding suggests that human IL-3 and/or GM-CSF may induce HSC exhaustion and accelerate hematopoietic maturation. We assessed the functional capacity of the engrafted human HSCs by performing serial BM transplantation in humanized mice (Fig. 3B). At all time points, the transfer of BM cells from NOGW mice to NOG mice resulted in a higher engraftment rate of human CD45^+^ cells than cell transfer from the other three strains (Fig. 3C). Although multilineage analysis of human hematopoietic cells showed no significant differences in leukocyte populations among the strains, significant levels of human platelets were detected 4 weeks after NOGW BM transplantation. This finding suggests that NOGW mice can more effectively preserve mature human megakaryocytes and progenitor cells than the other strains. In the BM of secondary recipients, the engraftment of CD45^+^ cells was the highest in the NOGW BM–transplanted group (Fig. 3D, E). This high engraftment rate may be attributed to the effective maintenance of human HSCs in NOGW mice (Fig. 3A). Conversely, secondary BM recipients who received BM transplantation from NOG-EXL and NOGW-EXL exhibited limited CD45^+^ cells and no CD34^+^CD38^+^ cells (Fig. 3D, E). These findings indicated that the long-term maintenance of human HSCs was more effective in NOGW mice than in human cytokine-expressing NOG-EXL and NOGW-EXL mice, despite the high rate of human myelopoiesis in these strains. Similar to this finding, previous studies have shown that NSG mice [21], which express three human cytokines—stem cell factor, GM-CSF, and IL-3—could not support the maintenance of transplanted human HSCs in the BM, owing to HSC exhaustion induced by the supraphysiological expression of these cytokines [22]. Nevertheless, these mice showed the long-term maintenance of mature lymphoid and myeloid cells, although most human HSCs were eliminated. Accordingly, maintenance of HSCs in the BM niche may not be necessary to reconstitute mature lymphoid and myeloid cells in humanized mice. Highly maintained lymphoid and myeloid progenitor cells with self-renewal capabilities may be responsible for this phenomenon in the cytokine-expressing humanized mice. However, further research is required to comprehensively clarify the implications of these findings and develop strategies to overcome the maintenance of any progenitor cells and HSC exhaustion induced by cytokine expression.

## Conclusion

Our study revealed that novel NOGW-EXL mice are highly suited for engraftment and multilineage differentiation of human hematopoietic cells following CD34^+^ human HSC transplantation in vivo, and NOGW mice demonstrated substantial maintenance of transferred human CD34^+^ cells in the BM. These models are useful for preclinical experiments in human hematological research.

### Electronic supplementary material

Below is the link to the electronic supplementary material.

Supplementary Material 1

## Data Availability

All data and materials generated and used in this study are able to be shared according to reasonable request to the corresponding author.

## Declarations

### ARRIVE guidelines

All the experiments in this work have been reported in line with the ARRIVE guidelines 2.0.

## Abbreviations

HSC: hematopoietic stem cell
NOG: NOD-scid IL-2rg^null^
SCID: severe combined immunodeficiency
IL: interleukin
NOGW: NOG-W41
NOG-EXL: NOG-hIL-3/GM-CSF Tg
BM: bone marrow
PB: peripheral blood
