# Supplementary material for: Improvement of multilineage hematopoiesis in hematopoietic stem cell-transferred c-kit mutant NOG-EXL humanized mice
Source: Stem Cell Res Ther. 2024 Jun 21;15:182. doi: 10.1186/s13287-024-03799-w (PMC11191313; doi:10.1186/s13287-024-03799-w)
Supplement: Supplementary file 1 — Supplementary Material 1 [file 13287_2024_3799_MOESM1_ESM.pdf]

Supplemental file

Title: Improvement of multilineage hematopoiesis in hematopoietic stem cell-transferred c-kit mutant NOG-EXL humanized mice

Authors

Ryoji Ito,[1*] Yusuke Ohno,[1] Yunmei Mu,[1,2] Yuyo Ka,[1] Shuko Ito,[1] Maiko Emi-Sugie,[1] Misa Mochizuki[1], Kenji Kawai[1], Motohito Goto,[1] Tomoyuki Ogura,[1] Riichi Takahashi,[1] Akira Niwa,[3] Tatsutoshi Nakahata,[1] Mamoru Ito[1]

Fig. S1

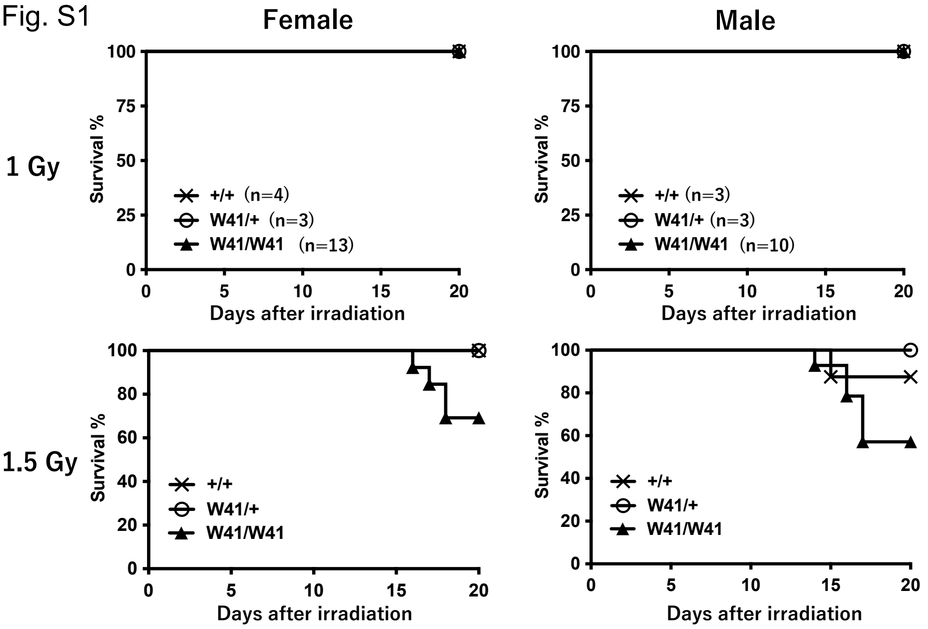

Figure S1. Irradiation sensitivity of NOG-W41 mice

Survival ratio of male and female of NOG (+/+), NOG-W41 heterozygous (W41/+), and homozygous (W41/W41) mice until 20-day period post 1 or 1.5 Gy x-ray irradiation.

Fig. S2

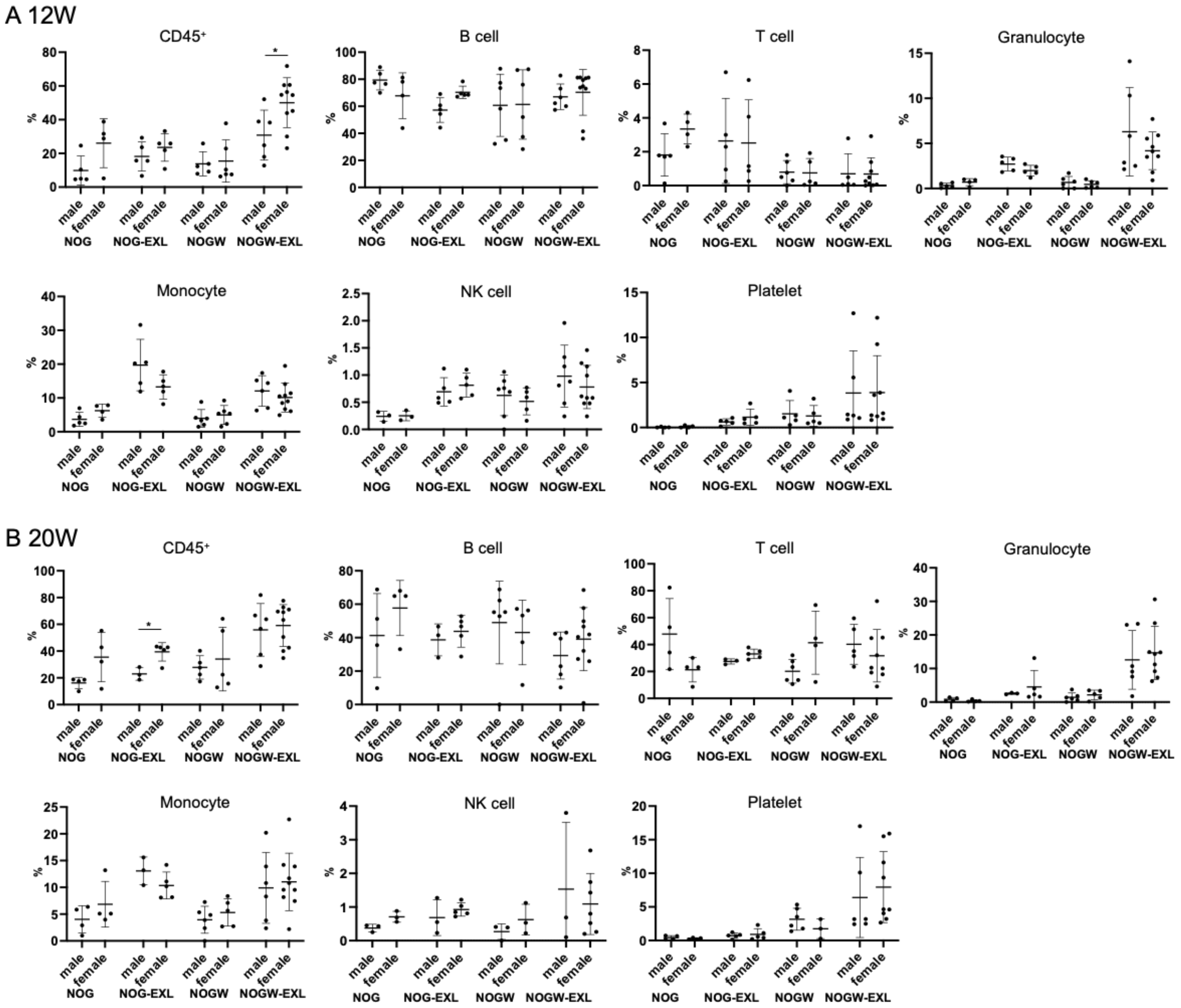

Figure S2. Comparison of human cell engraftment between male and female humanized mice

Frequencies of human $CD45^+$ cells, $CD19^+$ B cells, $CD3^+$ T cells, $CD66b^+$ granulocytes, $CD33^+14^+$ monocytes, $CD56^+$ NK cells, and $CD41^+$ platelets in the PB of humanized NOG, NOG-EXL, NOGW, and NOGW-EXL mice at 12 (A) or 20 (B) weeks after HSC transplantation. Male and female mice in Figure 2 were shown separately.

Fig. S3

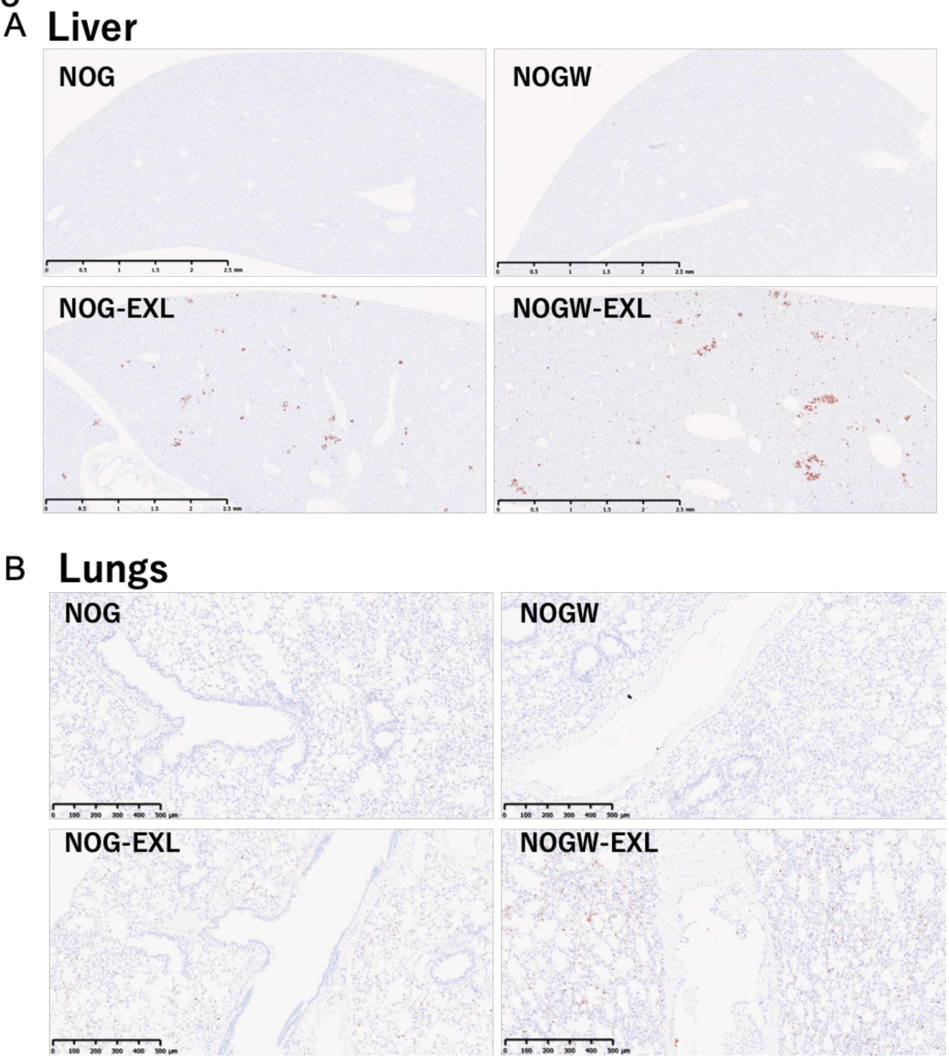

Figure S3. Tissue-resident human macrophages in humanized mice

Immunohistochemistry images of human CD68^{+} macrophages in the liver (A) and lungs (B) of humanized NOG, NOG-EXL, NOGW, and NOGW-EXL mice 20 weeks after HSC transplantation. Images are representative of three independent experiments.

Fig. S4

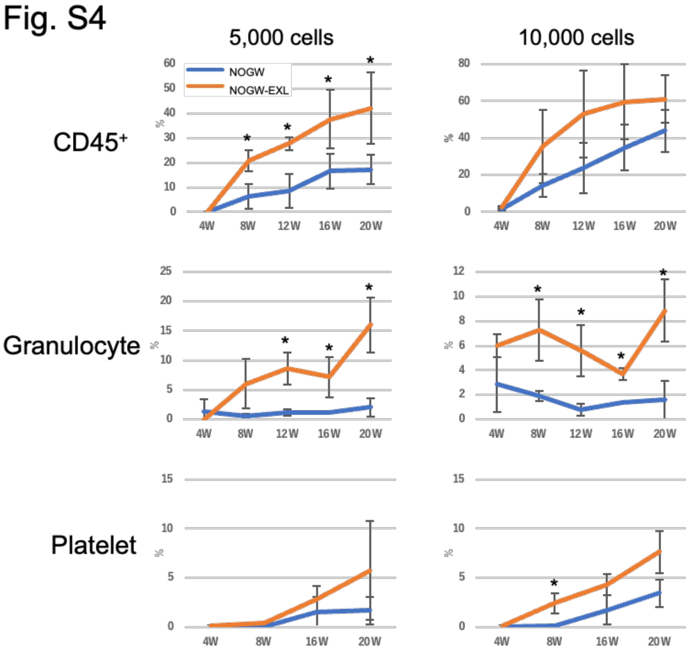

Figure S4. HSC limiting dilution assay in NOGW-EXL mice

Frequencies of human $CD45^{+}$ cells, $CD66b^{+}$ granulocytes, and $CD41^{+}$ platelets in the PB of humanized NOGW and NOGW-EXL mice 4–20 weeks after transplantation of 5,000 or 10,000 HSCs (n = 5). $^{*}p < 0.05$.

Table S1. List of the fluorescence-conjugated antibodies

Antibody	Fluorescence	Clone	Cat. no.	Company	Conc. (μg/ml)	Dilution	Isotype
mouse CD45	BUV395	30-F11	565967	BD Biocsience	200	1:500	Rat IgG2b
human CD45	BV510	HI30	304036	BioLegend	100	1:400	Mouse IgG1
human CD19	APC-Cy7	HIB19	302218	BioLegend	50	1:400	Mouse IgG1
human CD3	BV421	UCHT1	300434	BioLegend	50	1:300	Mouse IgG1
human CD66b	FITC	G10F5	305104	BioLegend	200	1:400	Mouse IgM
human CD33	PE	WM53	303404	BioLegend	200	1:400	Mouse IgG1
human CD16	APC	B73.1	360705	BioLegend	160	1:400	Mouse IgG2b
human CD14	BUV737	M5E2	564444	BD Biocsience	200	1:300	Mouse IgG2a
human CD56	PE-Cy7	HCD56	318318	BioLegend	100	1:400	Mouse IgG1
human CD38	PE	HB-7	356607	BioLegend	100	1:200	Mouse IgG1
human CD34	PE-Cy7	581	343515	BioLegend	100	1:200	Mouse IgG1
human CD41	PE-Cy7	HIP8	303718	BioLegend	100	1:500	Mouse IgG1
human CD201	APC	RCR-401	351905	BioLegend	100	1:250	Mouse IgG1
human CD45RA	APC-Cy7	HI100	304127	BioLegend	100	1:200	Mouse IgG2b
human CD10	BV421	HI10a	312217	BioLegend	20	1:200	Mouse IgG1
human Lineage cocktail (CD3, CD14, CD16, CD19, CD20, CD56)	FITC	UCHT1, HCD14, 3G8, HIB19, 2H7, HCD56	348801	BioLegend	Not specified	1:200	Mouse IgG1, IgG2a, IgG1, IgG1, IgG2b, IgG1
